# Electron Transfer from Haem to the Di‐Iron Ferroxidase Centre in Bacterioferritin

**DOI:** 10.1002/anie.202015965

**Published:** 2021-03-01

**Authors:** Jacob Pullin, Justin M. Bradley, Geoffrey R. Moore, Nick E. Le Brun, Michael T. Wilson, Dimitri A. Svistunenko

**Affiliations:** ^1^ School of Life Sciences University of Essex Wivenhoe Park Colchester Essex CO4 3SQ UK; ^2^ School of Chemistry University of East Anglia Norwich Research Park Norwich Norfolk NR4 7TJ UK

**Keywords:** electron transfer, ferritins, ferroxidase centre, metalloproteins, stopped-flow spectrophotometry

## Abstract

The iron redox cycle in ferritins is not completely understood. Bacterioferritins are distinct from other ferritins in that they contain haem groups. It is acknowledged that the two iron motifs in bacterioferritins, the di‐nuclear ferroxidase centre and the haem B group, play key roles in two opposing processes, iron sequestration and iron mobilisation, respectively, and the two redox processes are independent. Herein, we show that in Escherichia coli bacterioferritin, there is an electron transfer pathway from the haem to the ferroxidase centre suggesting a new role(s) haem might play in bacterioferritins.

## Graphical abstract

The haem in bacterioferritins has been shown before to provide an electron to the ferritin's mineral core—to reduce Fe^3+^ to water soluble Fe^2+^. This work shows that the haem can also provide an electron, over a distance of approximately 13 Å, to the di‐iron ferroxidase centre of the protein, in a redox process not yet known.

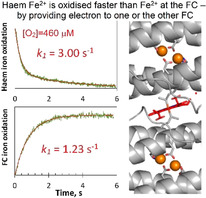

Ferritin is a ubiquitous iron storage protein found in almost all life forms. It binds ferrous ions, oxidises them to the ferric state, and deposits the oxidised iron inside the protein's shell as an insoluble mineral.^[1]^ The 24‐meric bacterioferritins (Bfrs) are distinct in the ferritin family as they can contain up to 12 haem B groups bound between two homo‐subunits.^[2]^ The haem is not needed for correct Bfr assembly and neither is it required for iron oxidation and mineralisation,^[3]^ although both the rate of the ferric mineral core formation and the amount of mineralised iron have been reported to depend on the haem content.^[4]^

The purified protein free of non‐haem iron (apo‐Bfr) is generally found with the haem in the Fe^3+^ state. The ferric haem iron oxidation state does not change during iron mineralisation in vitro under ambient conditions.[^3^, ^4^]

Bacterioferritins maintain iron homeostasis in cells by balancing two apparently independent redox reactions: iron mineralisation (Fe^2+^→Fe^3+^) and iron mobilisation via reduction of the mineralised Fe^3+^ to Fe^2+^ and its release to solution. While the di‐iron ferroxidase centre (FC) acts as the catalytic active site for the former redox process, the haem in Bfr appears to play a key role in the mobilisation of iron. The rate and extent of iron release from the mineral core, in the presence of external reductant, are significantly increased when haem is present.^[5]^ A role for the haem in iron mobilisation was demonstrated by Rivera and co‐workers who showed that, in *Pseudomonas aeruginosa*, a specific ferredoxin, Bfd, which is encoded next to the *bfr* gene,^[6]^ binds Bfr at a site close to the haem and passes an electron to the Bfr core, ultimately leading to mobilisation of Fe^2+^.^[7]^ Therefore, it appears the role of haem in Bfr is to transfer electrons from the cognate ferredoxin to the mineral core.

Herein, we demonstrate that ferrous haem can also provide an electron to the FC in a process not implicated so far in either iron mineralisation or iron mobilisation. The results allow us to postulate a new role for the haem in Bfr.

The haem group of *Escherichia coli* Bfr (EcBfr, Figure 1) may be reduced by sodium dithionite. When excess dithionite is removed by gel filtration (see Supporting Information), the haem group remains reduced during filtration and is only slowly oxidised under aerobic conditions (*t*_1/2_>2 h). This slow oxidation under air allows hours to perform experiments with apo‐EcBfr containing ferrous haem.

**Figure 1:**
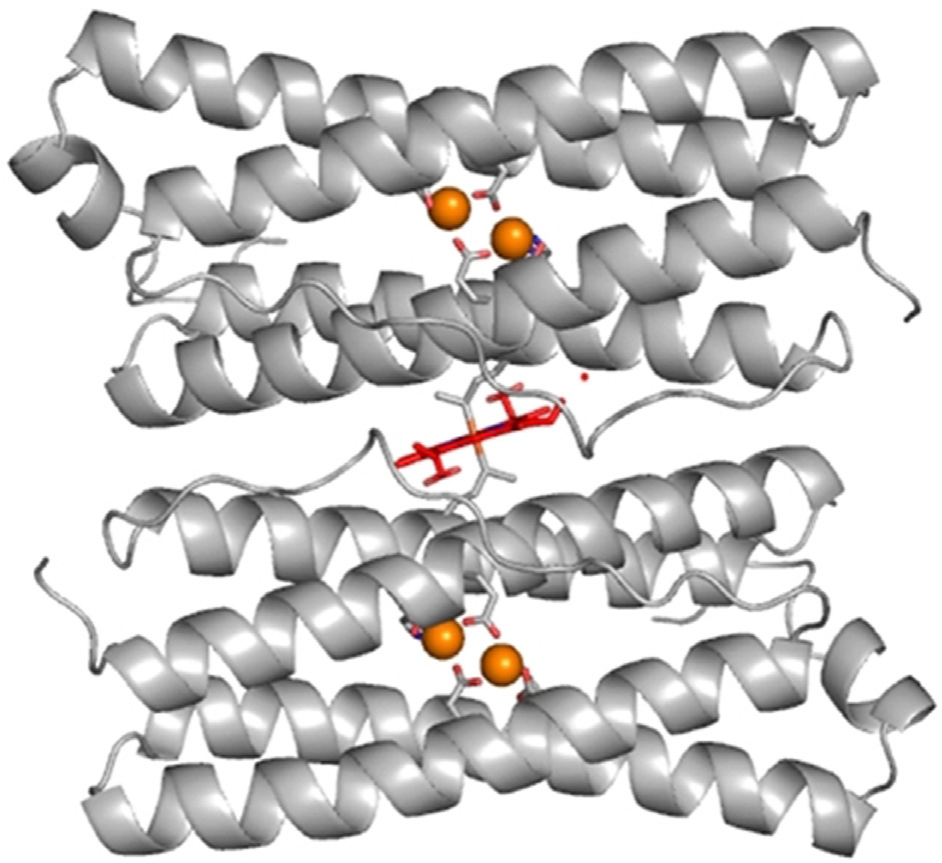
Haem B group (in red) at the interface of two subunits of EcBfr (PDB file 1BFR^[8]^). The identical subunits form a two‐fold symmetric haem binding site with Met52 of each subunit filling the two axial haem coordination positions. The metal ions in the FCs are indicated by orange spheres.

Figure 2 (blue trace) shows the UV/Vis spectrum of apo‐EcBfr with reduced haem to which external Fe^2+^ was added anaerobically in an amount to fill all the FCs. It is a typical Fe^2+^ haem spectrum.^[9]^ After oxygenated buffer was added, the spectrum changed rapidly to one typical for Fe^3+^ haem (orange trace), with a blue shift of the Soret band and the α‐ and β‐bands bleached. The underlying spectrum of ferric iron in the FC may also be discerned, having a broad band in the near UV and a tail that extends out to 600 nm. Similar spectral changes were observed when haem‐reduced EcBfr, anaerobically loaded with Fe^2+^, was mixed with H_2_O_2_ (Figure S1).

**Figure 2:**
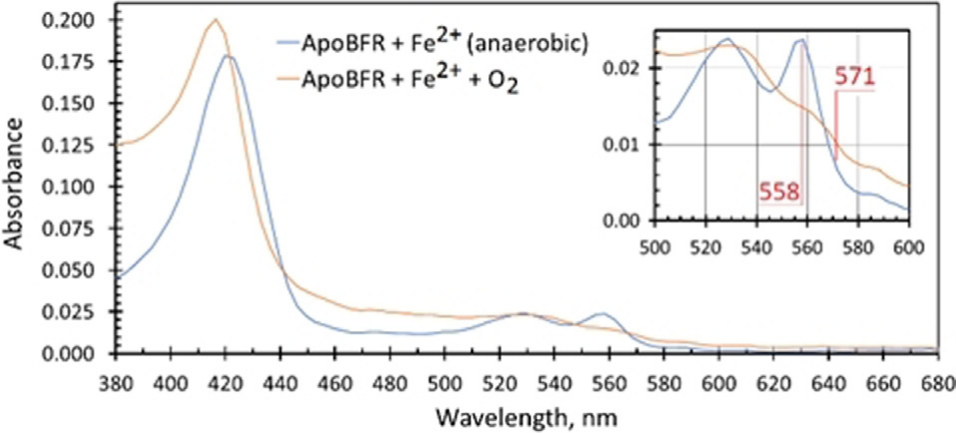
UV/Vis spectra of apo‐EcBfr (1 μm, pH 6.5), with reduced haem, loaded with 48 Fe^2+^ per 24mer before and after mixing with oxygenated buffer. After O_2_ addition (the spectrum was corrected for dilution) the α‐ and β‐bands of the haem are bleached (inset) and the Soret band is blue‐shifted. The two wavelengths used to measure absorbance difference change on haem iron oxidation (see Figures 3 and 4) are indicated in red in the inset.

In the kinetic experiments reported below, apo‐EcBfr containing haem in the ferrous state was mixed with Fe^2+^ ions in the presence of O_2_ or H_2_O_2_. In such experiments, Fe^2+^ ions must first bind to the FC after which oxidation can take place. Under the conditions used, the second‐order rate constant for Fe^2+^ binding to the FC has been reported to be 2.5×10^5^ m ^−1^ s^−1^ at 30 °C^[3]^ and thus at the [Fe^2+^] used, 48 μm, the pseudo‐first‐order constant for iron binding at this temperature is approximately 12 s^−1^. This rate constant will be lower at 25 °C, the temperature of the experiments reported below, but would remain some tenfold greater than the rate constant of Fe^2+^ oxidation by O_2_,^[10]^ and thus essentially all FC sites are filled by Fe^2+^ prior to oxidation using O_2_ as oxidant.

Figure 3 shows the results of stopped‐flow experiments carried out at two O_2_ concentrations (250 and 460 μm). Figure 3 A displays the time courses for Fe^2+^ oxidation monitored at 380 nm. These time courses may be fitted adequately by single exponentials (as shown), which yield rate constants (*k_1_*) of 0.86 and 1.23 s^−1^, in excellent agreement with earlier work.^[10b]^ Figure 3 B displays the optical changes monitored in the α region—the absorbance difference A_558_–A_571_ that reports on the haem redox state (see Figure 2 and Supporting Information for details). The addition of Fe^2+^ ions to apo‐EcBfr‐haem^2+^ in the presence of O_2_ leads to the oxidation of the haem. This can only occur through electron transfer (ET) from the haem to the FC as this is where the ultimate electron acceptor (O_2_) binds and oxidises the di‐ferrous FC. This conclusion is strongly supported by the observation that increasing O_2_ concentration from 250 to 460 μm leads to a rate enhancement in haem oxidation. The time courses in the α region change may be fitted by the sum of two exponentials, as shown, yielding the rate constants for the faster, dominant process of 2.0 and 3.0 s^−1^ for the lower and the higher O_2_ concentration, respectively, showing the same relative increase in rate constant on increasing O_2_ concentration as seen for iron oxidation at the FCs. The slower process (*k*_2_) identified in the fitting of the α region change has a lower first‐order rate constant of circa 0.3 s^−1^ that appears independent of O_2_ concentration (Figure 3). This process has not at present been assigned but may reflect some relaxation of the haem site following oxidation.

**Figure 3:**
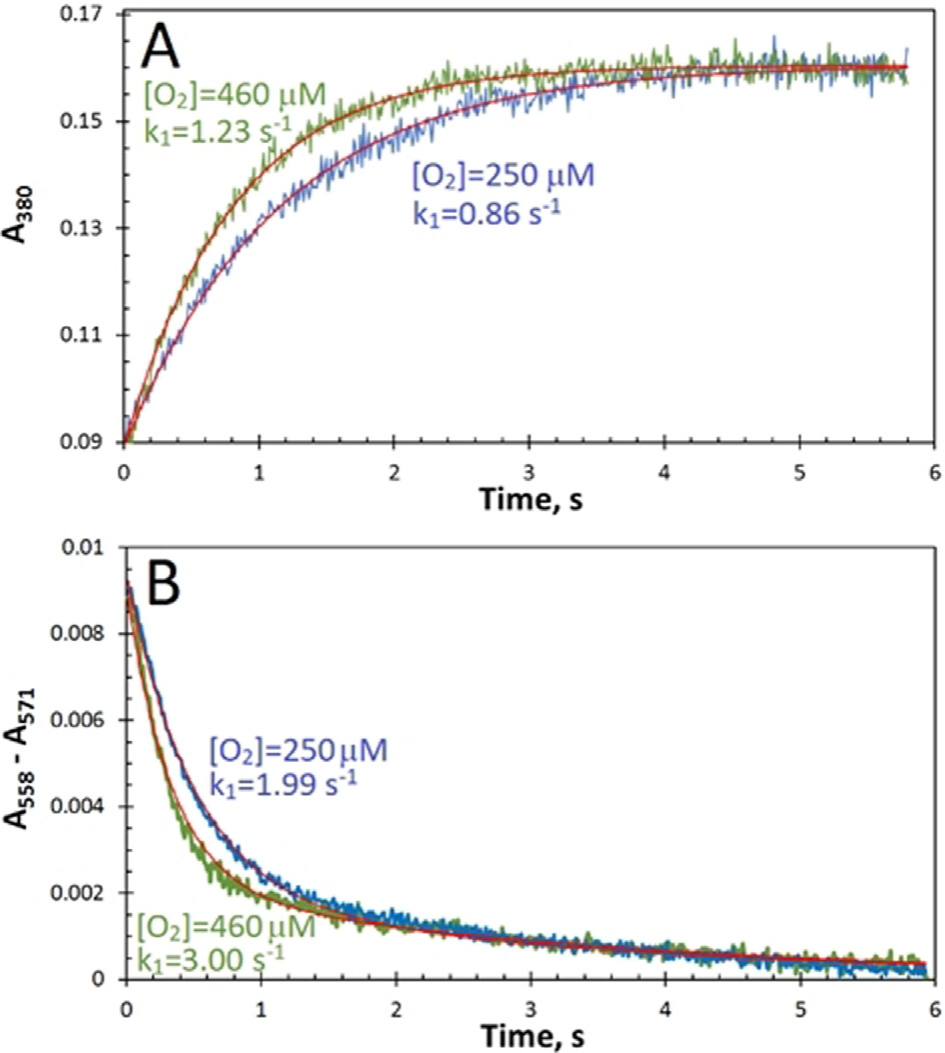
A) Time courses of Fe^2+^ oxidation in 1 μm EcBfr on loading with 48 μm Fe^2+^, monitored at 380 nm, at two different O_2_ concentrations. The fits to single exponentials ($A_{380}=\left(A_{0}+A_{1}\right)-A_{1}e^{k_{1}t})$ , with the *k*_1_ values indicated, are shown as red traces. B) Time courses of the oxidation of the haem groups monitored as the absorbance difference A_558_–A_571_, at the same two O_2_ concentrations. The time courses are fitted (red traces) with the double exponentials ($A_{558}-A_{571}=A_{0}+A_{1}e^{-k_{1}t}+A_{2}e^{-k_{2}t}$ ), with the faster rate constant values, *k*_1_, indicated. The slower rate constant *k*_2_ was found from the fitting to be 0.30 and 0.29 s^−1^ for 250 and 460 μm [O_2_], respectively.

A significant finding that follows from Figure 3 is that the rate constants for the faster process seen in the α region (haem iron oxidation) are greater by a factor of approximately 2 than those observed at 380 nm (oxidation of iron at the FCs) −1.99/0.86=2.3 and 3.00/1.23=2.4. This seemingly counterintuitive result may be understood by reference to Figure 1 in which it is seen that the haem group lies between two FCs of different subunits. Thus, the haem group has two routes through which it may be oxidised by the FCs and hence the rate constant for haem oxidation can be higher than the rate constant for the FC oxidation (possibly by a factor of 2 if ET is not rate limiting).

Replacement of Tyr25 and Tyr58, residues implicated in iron mineralisation^[11]^ and ET from Fe^2+^ in the central cavity to the FC,^[12]^ did not change the behaviour seen in Figure 3, thus demonstrating these residues are not involved in ET from the haem to the FC (Figures S2 and S3).

Hydrogen peroxide also oxidises Fe^2+^ ions once these are bound to the FC. We report in an accompanying paper that oxidation of Fe^2+^ ions is rapid (*k*=3.76×10^6^ m ^−1^ s^−1^).^[10b]^ Thus, at 50 μm H_2_O_2_ (see Figure S1), oxidation of the FC occurs at circa 190 s^−1^, approximately twentyfold faster than iron binding. Under these conditions, binding of Fe^2+^ to the FC becomes rate limiting. Figure 4 shows the time courses obtained in the stopped‐flow experiments in which the Fe^2+^ ions were mixed with solutions of apo‐EcBfr‐haem^2+^ containing H_2_O_2_ (the latter added to the protein immediately before loading a stopped‐flow syringe). The time course in Figure 4 A reports the oxidation of the Fe^2+^ ions at the FC, monitored at 380 nm, and that in Figure 4 B shows the oxidation of the haem group assessed from the A_558_–A_571_ difference change. Within the 1 s time span reported in Figure 4, the haem group is not oxidised by H_2_O_2_ in the absence of added Fe^2+^. Thus, on addition of Fe^2+^, the haem is oxidised via electron donation to the FC. The time courses in Figure 4 can be fitted by a single exponential, at 380 nm, and by the sum of two exponentials, in the α region. The rate constants of the faster processes in the two panels are close in value: 10.4 s^−1^ for Fe^2+^ oxidation at the FC and 12.3 s^−1^ for haem^2+^ oxidation. These values, determined at 25 °C, are consistent with the values for iron binding measured at 30 °C, that is, 12 s^−1^.^[3]^ The reason they are closely similar, and do not differ by a factor of about 2 as with O_2_, may also be understood in the light of the relatively slow filling of the FC with Fe^2+^. When using H_2_O_2_ as oxidant, as soon as a FC is filled with Fe^2+^, it is oxidised, and the probability of a given haem having two filled FCs in close proximity is small and thus there is little enhancement of the haem oxidation rate constant by providing two ET pathways, in contrast to oxidation by O_2_ which is slower than Fe^2+^ binding to the FC.

**Figure 4:**
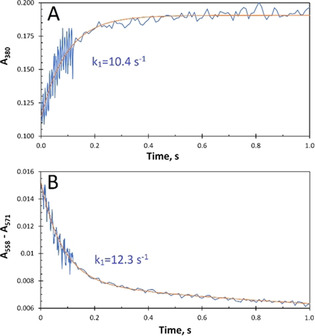
Time courses of Fe^2+^ oxidation at the FC A) and in the haem, monitored at the α region, B) after the solution of apo‐EcBfr, with haem^2+^ and H_2_O_2_ present, was mixed with ferrous iron (48 Fe^2+^ per 24mer). Concentrations in the mixture: [EcBfr]=1 μm, [H_2_O_2_]=50 μm (pH 6.5). The experimental traces were fitted (red traces) with a single exponential function $A_{380}=\left(A_{0}+A_{1}\right)-A_{1}e^{k_{1}t}$ (A), with the *k*_1_ values indicated, and a double exponent $A_{558}-A_{571}={A_{0}+A}_{1}e^{-k_{1}t}+A_{2}e^{-k_{2}t}$ (B), with the faster rate constant values, *k*_1_, indicated. The slower rate constant *k_2_* was found from the fitting to be 0.75 s^−1^. The faster and slower processes contribute 70 and 30 %, respectively, to the overall spectral change in (B).

The rate constant of the slower process in haem oxidation has apparently a higher value when H_2_O_2_ is used: *k*_2_=0.75 s^−1^ whereas *k*_2_ was 0.30 and 0.29 s^−1^ in the two O_2_ concentration cases. Above, we suggested that this process is likely to be associated with some relaxation of the haem site following iron oxidation. Comparison with the relaxation rate constant following oxidation by H_2_O_2_ is confounded by reaction of the di‐ferric site with excess of this oxidant. However, a comparison can be made with the slow process seen following oxidation by sub‐stoichiometric H_2_O_2_ where no such secondary reactions can take place (see Figure S6 in ref. [10b]). Here we observe a process with rate constant of approximately 0.1 s^−1^ that is independent of [H_2_O_2_]. Thus, the rate constants for relaxation using either O_2_ or H_2_O_2_ are quite close, the difference being due, we believe, to the differences in the time intervals over which the fitting was done.

The distances between the haem's iron and the two FC's irons are 13 and 14 Å (PDB file 3E1M^[13]^) allowing a rapid ET^[14]^ from the haem to the FC (as also demonstrated by photo‐induced ET in a Zn‐chlorin/Mn^2+^ construct of Bfr^[15]^). When oxidised FC encounters H_2_O_2_, free radicals are produced on the protein and could cause cellular damage.^[10b]^ When FC is reduced, it restores its capacity to scavenge H_2_O_2_ at a very high rate and without side production of reactive oxygen species.^[10b]^ Together with the fact of appropriate distance between the haem and the FCs, this suggests an additional role for the haem in Bfr, namely, the rapid reduction of the FC after it has been oxidised. Different in vitro procedures result in different haem content in the EcBfr 24mer, from under 1 to 12.[^3^, ^4^, ^11^, ^12^] The haem content in the purified protein was found to be 1 per 60 000 Da^[16]^ which is about 6 per EcBfr 24mer, and it seems likely that in vivo, the haem groups might occupy all available 12 sites and constitute an important means of maintaining a high H_2_O_2_ detoxification activity of EcBfr.

Scheme 1 depicts a branched pathway for ET through the haem. One route (marked A in yellow circle) suggested previously for *P. aeruginosa* BfrB,^[7]^ from Bfd (itself reduced by NADPH) to the haem, and then to the iron core reduces mineral iron to the soluble Fe^2+^ state. Through the second route B, as demonstrated for EcBfr in this work, electrons are transferred from the reduced haem to the FC. It remains to be elucidated how routes A and B are regulated, but the iron status of the cell (Fe^2+^ in solution and Fe^3+^ in the Bfr mineral core) and the extent of oxidative stress (concentration of H_2_O_2_) are likely to be important factors in determining the balance between them. It also remains to be determined if a mixed‐valence state of the FC is formed following transfer of a single electron from the haem to di‐ferric FC, and if so, how such a state further evolves.

**Scheme 1:**
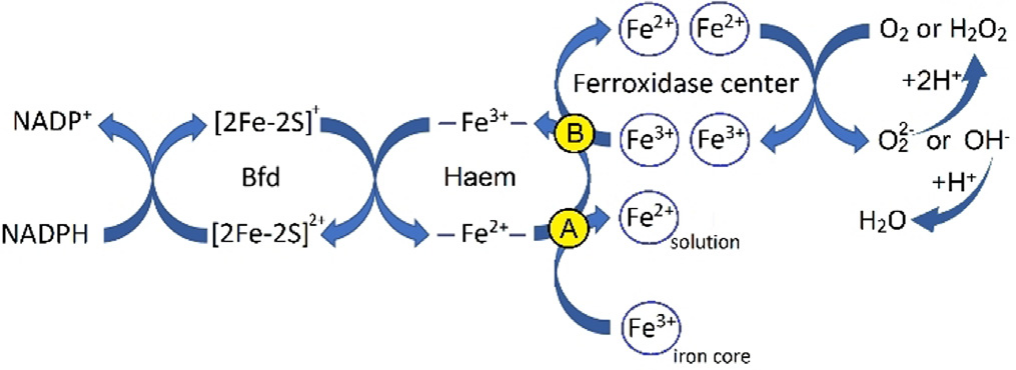
Fe^2+^ haem in bacterioferritins can reduce iron in two different redox reactions, both involving through‐protein ET: iron core reduction yielding ferrous ion into solution and oxidised FC reduction.

In conclusion, the discovery of direct haem to FC ET in EcBfr prompts a new line of experimental enquiry to elucidate the mechanism of H_2_O_2_ detoxification. Reduced haem appears to be not only the source of electrons to mobilize iron from the core but also a source to rapidly restore FCs to the ferrous state which can rapidly scavenge H_2_O_2_.

## Conflict of interest

The authors declare no conflict of interest.

## Supporting information

As a service to our authors and readers, this journal provides supporting information supplied by the authors. Such materials are peer reviewed and may be re‐organized for online delivery, but are not copy‐edited or typeset. Technical support issues arising from supporting information (other than missing files) should be addressed to the authors.

Supplementary
