# Supplementary material for: Electron Transfer from Haem to the Di‐Iron Ferroxidase Centre in Bacterioferritin
Source: Angew Chem Int Ed Engl. 2021 Mar 1;60(15):8376–9. doi: 10.1002/anie.202015965 (PMC8048850; doi:10.1002/anie.202015965)
Supplement: Supplementary file 1 — Supplementary [file ANIE-60-8376-s001.pdf]

Angewandte Chemie
Eine Zeitschrift der Gesellschaft Deutscher Chemiker

Supporting Information

Electron Transfer from Haem to the Di-Iron Ferroxidase Centre in Bacterioferritin

Jacob Pullin, Justin M. Bradley, Geoffrey R. Moore, Nick E. Le Brun, Michael T. Wilson, and Dimitri A. Svistunenko**

anie_202015965_sm_miscellaneous_information.pdf

Table of Contents

Experimental Procedures . . . . page 2
EcBfr expression; haem content determination . . . . page 2
Reduction of haem; removal of dithionite . . . . page 2
Static UV-vis spectrophotometry . . . . page 2
Stopped flow experiments . . . . page 2
Kinetics of iron oxidation - at the FC and at the haem . . . . page 2
Excel Solver fitting . . . . page 3
Supporting figures . . . . page 3
Figure S1 . . . . page 3
Figure S2 . . . . page 3
Figure S3 . . . . page 4
References . . . . page 4
Author contributions . . . . page 4

Experimental Procedures

EcBfr expression; haem content determination

Wild type and variant EcBfr proteins were prepared as previously described.[1] Non-haem iron was removed by treatment with sodium dithionite and bipyridyl, as previously described.[2] The monomeric concentrations of EcBFR were determined using the following ε_{280} values, all in units of M^{-1} cm^{-1}: 33 000 (WT),[3] 25 585 (Y25F),[1a] 24 600 (Y58F).[1a]
Haem content of proteins was determined, following non-haem iron removal, using the haem Soret absorbance intensity (ε_{418} = 107 000 $M^{-1}cm^{-1}$,[4]) and found to be 1.0–1.5 haem/BFR for all variants.

Reduction of haem; removal of dithionite

Haem was reduced by addition of a small excess of sodium dithionite which was then removed by passage through a PD-10 gel filtration column.

Static UV-vis spectrophotometry

‘Static’ UV-vis spectra of the protein before and after O_2 was added (Figure 2) were obtained on a Cary UV-vis spectrophotometer (Agilent Technologies).

Stopped flow experiments

An Applied Photophysics (Leatherhead, UK) SX20 stopped-flow spectrophotometer, equipped with a diode array detection systems, with the reaction cell maintained at 25 ± 0.1 °C, was used. Ferrous ammonium sulfate, at a concentration required to fill all FCs, was added to a 1 µM apo-EcBfr with reduced (Fe^{2+}) haem solution that also contained either O_2 (two concentrations of O_2 were analysed) or H_2O_2. In the O_2-driven oxidation experiments, the lower O_2 concentration, 250 µM, was the ambient O_2 concentration in the protein and the Fe^{2+} solutions as determined from O_2 solubility at the atmospheric temperature and pressure on the day.[5] The higher O_2 concentration in the mixture analysed, 460 µM, was achieved by mixing an air equilibrated protein solution (1 µM apo-EcBfr with reduced haem) with an O_2 saturated (to 670 µM) Fe^{2+} solution.

Kinetics of iron oxidation - at the FC and at the haem

The time course of added iron oxidation at the FCs was evaluated by the absorbance increase at 380 nm. The haem iron oxidation over time was assessed from the changes in the α- and β-bands of haem optical spectra: the time courses were constructed by taking the difference in absorbance at two wavelength (558 nm and 571 nm) that are close and on opposite sides of the isosbestic point of spectral changes associated with oxidation of ferrous to ferric haem. In doing this, interference from the broad underlying

Fe^{3+} spectrum is eliminated and noise due to time variation in the baseline, which can be significant when monitoring very small absorbance changes over time, is also minimised.

Excel Solver fitting

Fitting experimental kinetic dependences with analytical functions was performed with Excel Solver routine.

Supporting figures

Figure S1

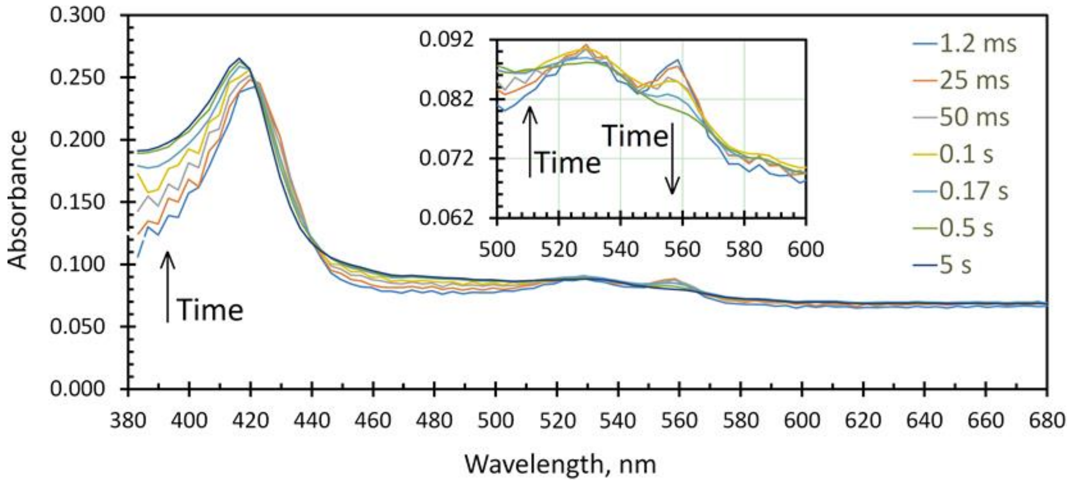

Figure S1. UV-vis spectra of the WT apo-EcBfr (1 µM, pH 6.5), with reduced (Fe^{2+}) haem, loaded with 48 Fe^{2+} per 24mer in the course of a stopped-flow experiment when mixing with 50 µM H_2O_2 (all concentrations are in the mixture).

Figure S2

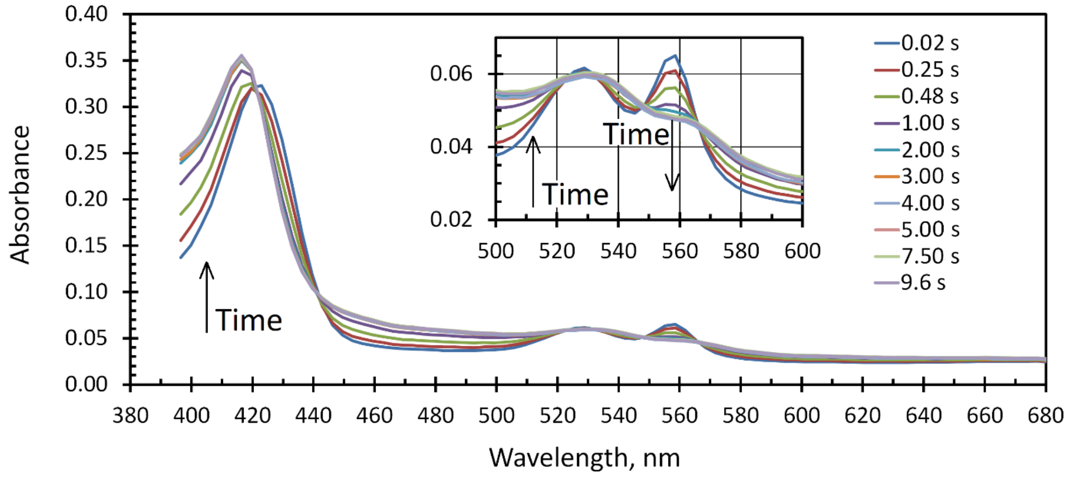

Figure S2. UV-vis spectra of 2.1 µM Y25F apo-EcBfr, with reduced (Fe^{2+}) haem, in the course of a stopped-flow experiment when mixing with Fe^{2+} (48 Fe^{2+} per 24mer) under ambient oxygen conditions (all concentrations are in the mixture).

Figure S3

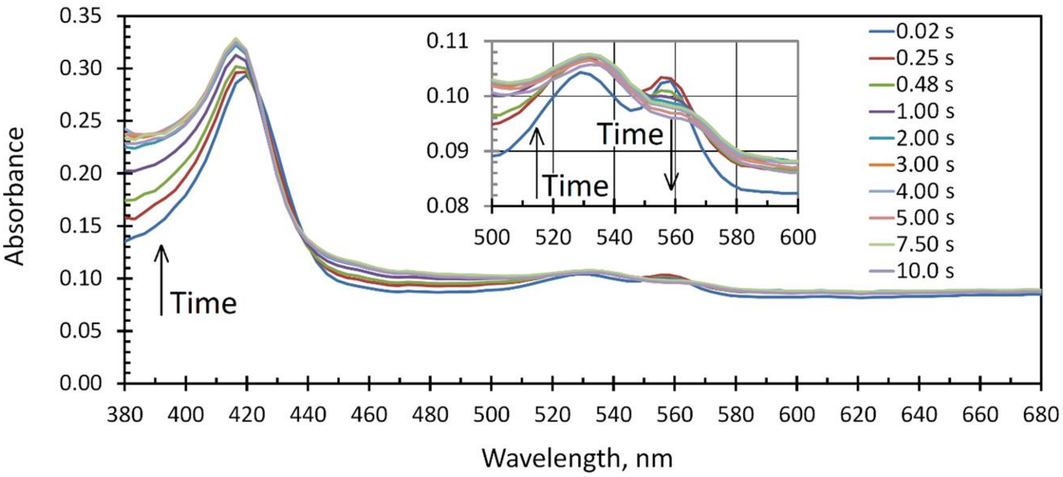

Figure S3. UV-vis spectra of 2.1 µM Y58F apo-EcBfr, with reduced (Fe^{2+}) haem, in the course of a stopped-flow experiment when mixing with Fe^{2+} (48 Fe^{2+} per 24mer) under ambient oxygen conditions (all concentrations are in the mixture).

References

[1] a) J. M. Bradley, D. A. Svistunenko, T. L. Lawson, A. M. Hemmings, G. R. Moore, N. E. Le Brun, *Angew. Chem. Int. Ed. Engl.* **2015**, *54*, 14763–14767; b) T. L. Lawson, A. Crow, A. Lewin, S. Yasmin, G. R. Moore, N. E. Le Brun, *Biochemistry* **2009**, *48*, 9031-9039.

[2] E. R. Bauminger, P. M. Harrison, D. Hechel, I. Nowik, A. Treffry, *Biochim. Biophys. Acta* **1991**, *1118*, 48-58.

[3] a) A. Crow, T. L. Lawson, A. Lewin, G. R. Moore, N. E. Le Brun, *J. Am. Chem. Soc.* **2009**, *131*, 6808-6813; b) X. Yang, N. E. Le Brun, A. J. Thomson, G. R. Moore, N. D. Chasteen, *Biochemistry* **2000**, *39*, 4915-4923.

[4] M. R. Cheesman, N. E. le Brun, F. H. Kadir, A. J. Thomson, G. R. Moore, S. C. Andrews, J. R. Guest, P. M. Harrison, J. M. Smith, S. J. Yewdall, *Biochem. J.* **1993**, *292 (Pt 1)*, 47-56.

[5] J. K. Gundersen, N. B. Ramsing, R. N. Glud, *Limnol. Oceanogr.* **1998**, *43*, 1932-1937.

Author Contributions

J. Pullin - performed the experiments and data analysis, wrote original draft - equal
J. Bradley - performed the experiments, wrote parts of the initial draft - equal
G. Moore – edited the manuscript - equal
N. Le Brun – funding acquisition, edited the manuscript - equal
M. Wilson – conceived the study, performed the experiments and data analysis, wrote original draft, edited the final version– lead
D. Svistunenko - funding acquisition, performed data analysis, made the figures, wrote the final version of the manuscript – lead
